# Impact of Carbohydrate Ingestion on Cognitive Flexibility and Cerebral Oxygenation during High-Intensity Intermittent Exercise: A Comparison between Maple Products and Usual Carbohydrate Solutions

**DOI:** 10.3390/nu11092019

**Published:** 2019-08-28

**Authors:** Olivier Dupuy, Jonathan Tremblay

**Affiliations:** 1 Laboratoire MOVE (EA 6314), Faculté des Sciences du Sport, Université de Poitiers, 86000 Poitiers, France; 2 École de kinésiologie et des sciences de l’activité physique, Faculté de Médecine, Université de Montréal, Montréal, QC H3T 1J4, Canada

**Keywords:** maple products, cognitive performance, switching task, cerebral oxygenation

## Abstract

Background: The aim of this study was to compare the effects of carbohydrate (CHO) drinks (6% per volume) sweetened with maple (syrup or sap) to a commercial sports drink, glucose, and a control solution (water) on cognitive flexibility during high-intensity intermittent exercise. Methods: Eighty-five active men completed six 3-min bouts at 95% of their maximal aerobic power on a stationary bike, with 3 min of passive rest between efforts. Subjects were randomly allocated to an ingestion condition. Following each exercise bout, subjects ingested 166 mL of the experimental solution, drinking a total of 1 L of the same solution throughout the experimentation. Cognitive flexibility was measured using reaction time and accuracy on the Stroop task. The cognitive task was performed a total of 10 times, including 15 and 30 min post-exercise. Glycemia and cerebral oxygenation were also measured at each time point. Statistical analyses were performed using a two-way ANOVA (Condition × Time) with repeated measures. Results: The ingestion of maple products and the commercial sports drink led to a lesser increase in glycemia than glucose ingestion. CHO ingestion, when compared to water, induced a slight reduction in reaction times on the cognitive task, especially in the switching trials. CHO ingestion had no impact on cerebral oxygenation. Conclusions: This study shows that CHO ingestion, regardless of its type, tends to improve cognitive performance throughout exercise, especially during difficult cognitive tasks.

## 1. Introduction

Carbohydrate (CHO) intake during exercise is known to improve both short intermittent and prolonged continuous physical performance [1,2]. Multiple mechanisms have been suggested to be responsible for this improvement [2,3], which mainly depend on the exercise duration. Some are related to energy availability, while others are associated with afferent inputs in the central nervous system. Indeed, the ingestion of CHO before and during exercise maintains the plasma glucose concentration and allows for an increased rate of CHO oxidation, often leading to an improvement in exercise performance. However, the impact of glucose ingestion on performance could also be mediated by the central nervous system [4]. Given that CHO does not appear to be the limiting factor for performances lasting less than one hour, a greater contribution of the centrally-mediated mechanisms appears relevant in these efforts. This hypothesis was strengthened by mouth-rinsing studies reporting an increased activation of the brain’s pleasure and motivation pathways related to the presence of a CHO solution in the mouth [5,6,7]. Since cognitive processes play a major role in physical performance, a possible explanation for the physical improvements reported after CHO ingestion could be mediated by cognitive improvements [2,4]. Several sports require strong executive functions, such as cognitive flexibility and inhibition, in order to make timely decisions and perform. However, the impact of CHO intake on cognitive inhibition and flexibility during exercise has not been thoroughly investigated, and the available results remain unclear [8,9].

Nutrition is known to play a role in cognitive and cerebral function in both young and old individuals [4,10]. In young adults, studies suggest that acute CHO supplementation and the resulting increase in blood glucose concentration improves cognitive performance in attentional tasks [11,12,13] and when executive processes are required [14,15]; these benefits were reported even in non-fasting individuals. Conversely, hypoglycemia negatively affects brain activity and cognitive performance [4]. For example, impaired glucose regulation is associated with poorer episodic memory performance [16]. The impact of CHO on cognitive performance was mechanistically supported by several neurophysiological studies showing that glucose ingestion has a direct impact on brain activity. As proposed in fMRI [5] and studies using electroencephalography [6], oral glucose receptors could activate the brain by improving arousal and motivation after glucose ingestion. Vascular mechanisms could also be involved since cerebral oxygenation seems to be improved after glucose ingestion [17]. It is worth noting, however, that most studies reporting a positive effect on brain activity and cognitive performance were completed at rest, without exercise.

Many studies have reported the effect of glucose ingestion on cognitive performance during or after physical exercise [4,8,18]. Collardeau et al. [19] found that the consumption of a 5.5% CHO-electrolyte solution before and during a 100-min run improved reaction times of triathletes during a complex cognitive test at the end of that run. Furthermore, [20] have reported that the ingestion of a 6.4% CHO solution was associated with improved visual choice reaction time during simulated squash play. More recently, Pomportes et al. [9,21] reported that glucose ingestion or glucose mouth-rinsing was associated with an improved performance in the Simon task during submaximal exercise. However, other studies have also reported that CHO ingestion had no impact on motor skills or cognitive performance (see the review by Baker et al. [8]). Several methodological causes can explain these contradictory results. First, it seems that the cognitive processes and tasks explored in these reports are not all sensitive to CHO availability. The type of CHO also appears to influence the results in something of a dose–response manner [8,22]. Finally, the large variety of protocols and statistical approaches to assess cognitive performance also contribute to the conflicting results in the literature.

Most reports have used glucose solutions or commercial sports drinks but rarely natural or unprocessed products. Some studies have recently assessed the impact of guarana on cognitive performance and reported that it could enhance cognitive performance at rest or during exercise [9,21,23]. Also, the impacts of plant products and some of their bioactive compounds, such as polyphenols, ginseng, ginkgo biloba and others, have received growing interest in the last few years [8,18]. Maple products such as maple syrup and maple water (sap from maple trees), which mainly contain sucrose but also many other bioactive ingredients [24], could also potentially impact cognitive performance through the activation of taste receptors in the mouth or through various metabolic pathways. For example, maple syrup contains various polyphenols (phenolic acids, flavonols, lignans, etc.) which are known for their antioxidant properties, but have also been suggested to improve cognition [4,18]. Recent work has shown that a maple syrup extract could reduce neuroinflammation in microglial and neuronal cells in *Caenorhabditis elegans* with induced neurotoxicity [25]. To the best of our knowledge, no studies have so far investigated the effect of maple products on physiological and cognitive responses during exercise.

Some researchers have hypothesized that supplying glucose to the brain would affect performance more under conditions of high cognitive demand and physical effort [8,26]. Messier [16] reported that CHO ingestion appears to have a greater impact when the task difficulty increases during memory tasks or when attention is divided between two tasks. Based on this evidence, the current study attempts to compare the impact of different types of ingestion (water, glucose, a commercial sports drink, maple syrup, and maple water) before and during high-intensity intermittent exercise, all set at the same CHO content (60 g/L), on cognitive flexibility, which is known to be a very demanding executive task.

## 2. Materials and Methods

### 2.1. Subjects

The experiment was conducted on 85 recreationally active males who gave their informed written consent to participate in this study, which was approved by the University of Montreal ethics committee on health research (#17-030-CERES-P). All subjects were moderately active (4–6 h∙week^−1^) and had normal plasma glucose concentrations after a 12 h fast as well as 120 min after ingestion of 75 g of glucose in 300 mL of water (Table 1).

### 2.2. Experimental Protocol

In the first visit, following a thorough briefing, all participants signed a written statement of informed consent and performed an oral glucose tolerance test (OGTT). In a second visit, participants were familiarized with the cognitive tasks to learn the response procedure and attempt to reduce their error rate. They also completed a maximal continuous graded exercise test on a cycle ergometer to determine their peak power output (PPO) and maximal oxygen consumption (VO_2max_). In the third visit, the participants were invited to perform the experimental protocol beginning with a standardized warm-up followed by the high-intensity intermittent exercise (HIIE) protocol. The warm-up began with 10 min at 50% of PPO, followed by 6 × 10 s sprints at 95% of PPO, interspersed with a minute of active recovery. The HIIE protocol consisted of a series of 6 × 3-min cycling bouts, performed at 95% of PPO and interspersed by 3 min of passive recovery. Ratings of perceived exertion (RPE, [27]) were systematically measured after each bout of exercise. Using the modified Borg scale, ratings ranged from 1 to 10, with 10 being maximal perceived exertion.

**Figure 1:**
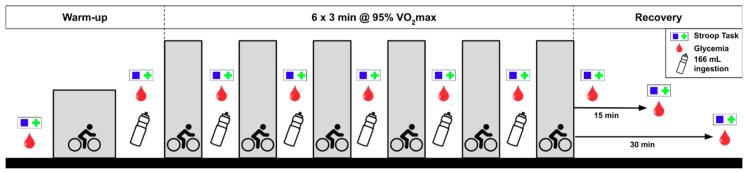
Experimental protocol.

During the third visit, participants performed a computerized version of the Stroop Color Word Test with the measurement of cerebral oxygenation before, during (recovery intervals), and at 15 and 30 min after HIIE. This procedure was presented in a previous study [28]. The perceived difficulty of the cognitive task was systematically measured with the DP-15 scale [29,30] following each cognitive assessment. This scale was used previously in several reports [28,31,32]. The subjects were required to provide a rating for the Stroop task ranging from 1 to 15 (where 1 corresponds to “extremely easy”; 6 to “easy”; 10 to “difficult”; 15 to “extremely difficult”). Participants had to refrain from intense exercise for the 2 days before each experiment. Alcohol and caffeine intake were forbidden for at least 12h before experimentation, and the subjects were instructed to maintain their habitual diet. All experimental sessions took place in a room kept at a constant temperature (21 °C) and humidity (45%). The experimental design is summarized in Figure 1.

### 2.3. Placebo and CHO Solutions

Five solutions were administered in this study: water (control), glucose, a commercial sports drink (CSD), diluted maple syrup, and concentrated maple water. The CHO content of the CHO solutions (glucose, CSD, maple syrup, and maple water) was standardized to 60 g/L. Maple water was provided by the Québec Maple Syrup Producers and had been concentrated to 60 g/L of CHO by reverse osmosis. Maple syrup and glucose were diluted in water at the same CHO concentration as in the commercial sports drink (60 g/L). All subjects were randomly assigned to one of these solutions which were administered (166 mL, total of 1 L) in an opaque bottle before each bout of 3 min at 95% of PPO, as shown in Figure 1.

### 2.4. Measurements

#### 2.4.1. Plasma Glucose Concentration

Glycemia was measured using Aviva’s AccuCheck blood glucose monitor before and during recovery intervals after HIIE bouts (immediately after the completion of each cognitive task), and 15 and 30 min after the last bout.

#### 2.4.2. Peak Power Output and Maximal Oxygen Consumption

Maximal oxygen consumption (VO_2max_) was measured using open-circuit spirometry (Ultima™ CardiO2^®^, MGC Diagnostics, Saint Paul, MN, USA) on a cycle ergometer (Excalibur Sport, Lode BV, Groningen, Netherlands). An incremental protocol was initiated with a power output set at 125 W for 5 min and increased by 30 W every 3 min until participant exhaustion. The power output of the last completed stage was considered as the peak power output (PPO; in W).

#### 2.4.3. Cognitive Task

Cognitive performance was assessed by the computerized modified Stroop task described previously [28,33] and briefly below. The assessment is based on the modified Stroop Color Word Test [34] and is known to specifically assess executive functions under the control of the prefrontal cortex. Executive functions generally refer to the “high-level” control and regulation of cognitive processes such as planning, inhibiting routine behavior or updating working memory.

As shown in Figure 1, the cognitive task was first performed before the standardized warm-up (baseline), after 15 min of a quiet period in the laboratory and 2–3 min after the end of the warm-up. During the HIIE, the participants completed the cognitive task 30 s after completing each bout of exercise. During the recovery period, following the last bout of exercise, the participants completed the cognitive task at 15 and 30 min following exercise. Every time, the participants remained sitting on the cycle ergometer throughout the cognitive task.

The modified computerized Stroop task required the participant to select the color (blue or green) of items displayed on a screen. Participants provided their answers by pressing USB-controlled response buttons fixed on the handlebars of the cycle ergometer. Participants were instructed to respond as quickly as possible while minimizing errors. The familiarization block allowed participants to learn the assignment of the response buttons. Typically, this Stroop task has four conditions that range from simple (reading: see BLUE in blue ink, say BLUE) to complex (see BLUE in green ink, but report the color of the ink: green). In this study, we selected the most complex condition of the Stroop test (switching/interference) to contrast with reading. In this semantic interference condition, participants had to either choose the ink color of the color-word or to read the word. This condition also included a switching task, where for 25% of the trials, a square appeared on the screen instead of a cross before the word was presented, and participants were then required to read the color-word instead of naming the color of the ink. The reading trials appeared randomly throughout the block. These instructions were counterbalanced between participants. During the familiarization, participants completed 2 × 60 practice trials. During both familiarization and practice trials, visual feedback (“Error!”) was displayed on the screen when an incorrect response was selected. Each of the 10 blocks of the experimental sessions contained 30 trials with a blank screen between the trials. To reduce the learning effect, practice trials (*n* = 20) were performed before the first experimental block. Reaction time (ms) and error (accuracy, %) were recorded. This procedure has been used previously in several studies [28,35,36,37]. Based on the evidence that some cognitive tests are not sensitive to CHO ingestion, we calculated the total reaction time (all trials combined), the reaction time for the congruent trials (read the word) and incongruent trials (provide the color of the ink), and the reaction time for the switching trials (instructions differ from one trial to another).

### 2.5. Cerebral Oxygenation

Changes in oxyhemoglobin (HbO_2_) and deoxyhemoglobin (HHb) concentrations were measured by multichannel, near-infrared continuous-wave spectroscopy (NIRS) (Artinis, Octamon, Einsteinweg, The Netherlands), using wavelengths of 830 and 690 nm which are more sensitive to HbO_2_ and HHb, respectively. Combining the multispectral measurements with known extinction coefficients of hemoglobin, variations in HbO_2_ concentration were computed using the modified Beer–Lambert law and a path-length factor based on the age of the participant. Two arrays of 4 sources and 1 detector were tightly secured on the prefrontal cortex with a tensor bandage wrapped around the forehead, taking sufficient care to ensure that there was no interference of background light and to limit movement during the cognitive task. Two probes (one on each hemisphere) were arranged with one central, anterior–posterior row of four emitters per hemisphere. Four channels were placed strategically away from the emitters. The two probes were placed symmetrically over the lateral prefrontal cortex, and the most anterior and most ventral emitter–detector pair of each probe was placed on Fp1/Fp2 using the 10/20 system. Fp1 and Fp2 regions have been found to correspond to the superior and medial frontal gyri [38]. This method and setup have already been used successfully in our previous research projects [17,33]. The source–detector pairs were combined into four different approximate regions of interest (ROIs) that do not refer exactly to the underlying brain regions and cover the anterior dorsolateral prefrontal cortex (Broadman aera 9, 10 and 46), the posterior dorsolateral prefrontal cortex (Broadman aera 6 and 4), the anterior ventrolateral prefrontal cortex (Broadman aera 10, 45 and 46) and the posterior ventrolateral prefrontal cortex (Broadman aera 4, 6, and 44) for both hemispheres. Variables of interest were relative changes in concentration of HbO_2_, HHb, and total hemoglobin (ThB) compared to the baseline [17,33], because continuous-wave technology does not allow the quantification of absolute concentration due to the incapacity of measuring optical path lengths [39,40]. Baseline measures were taken from the last of 5 min at rest on the cycle ergometer before block 1. Cerebral oxygenation was measured continuously throughout the experimental protocol (rest, exercise and recovery), and the cognitive task was labelled in order to extract the data during its completion.

### 2.6. Statistical Analyses

Data are presented as mean ± standard deviation. The normal Gaussian distribution of the data was verified by the Shapiro–Wilk test. All variables met this underlying hypothesis. The effects of the solutions and comparisons over the course of the experiment were made using a two-way (ingestion × time) repeated measures ANOVA. Multiple comparisons were made using Tukey’s honest significant difference; the magnitude of the differences using the Hedge’s g effect size (ES) and the percentage change from baseline measures were reported. The significance level was set at *p* < 0.05 for all analyses. The magnitude of the difference was considered very small (<0.2), small (0.2 < ES < 0.5), moderate (0.5 < ES < 0.8), large (0.8 < ES < 1.2) and very large (ES > 1.2) [41].

## 3. Results

Characteristics of the subjects in each experimental group are presented in Table 1.

**Figure 2:**
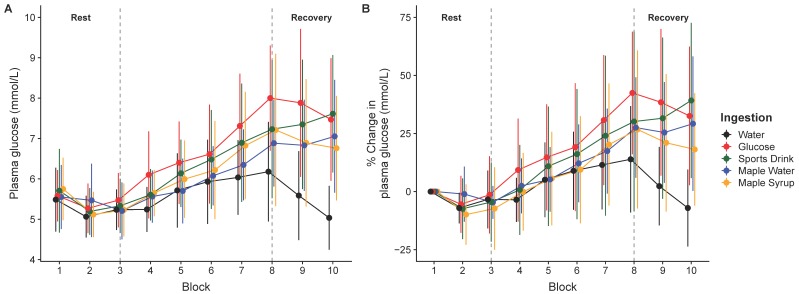
Plasma glucose concentration (**A**) and percentage of change (**B**), at baseline, over the high-intensity intermittent exercise (HIIE) protocol and during recovery (mean ± SD).

The glycemic response during the HIIE protocol is shown in Figure 2. The ANOVA (ingestion × time) revealed a main effect of time (F_(4252)_ = 59.0, *p* < 0.0001) and a significant ingestion × time interaction (F_(15,252)_ = 3.2, *p* < 0.0001). When glucose was ingested, post-hoc analyses show a significant increase in plasma glucose concentration from the baseline, starting at the 4th exercise bout (block 6; 5.5 ± 0.7 mmol·L^−1^), and peaking after the last bout (block 8; 8.0 ± 1.3 mmol·L^−1^), while this was observed later for maple products (from the 5th bout for maple syrup and from the 8th bout for maple water) and CSD (5th bout). There was no significant change in glycemia throughout the HIIE protocol when water was ingested.

**Figure 3:**
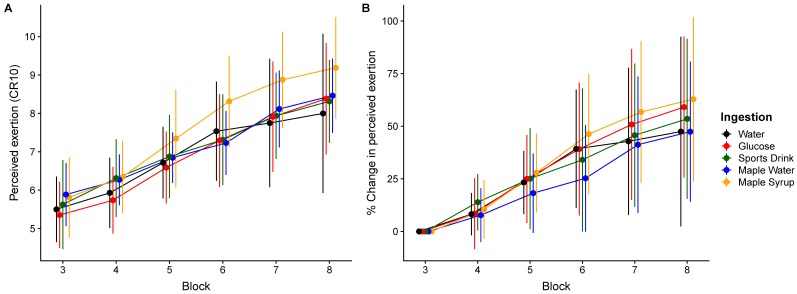
Evolution of ratings of perceived exertion (RPE) (**A**) and percentage of change (**B**) over the high-intensity intermittent exercise protocol (mean ± SD).

**Figure 4:**
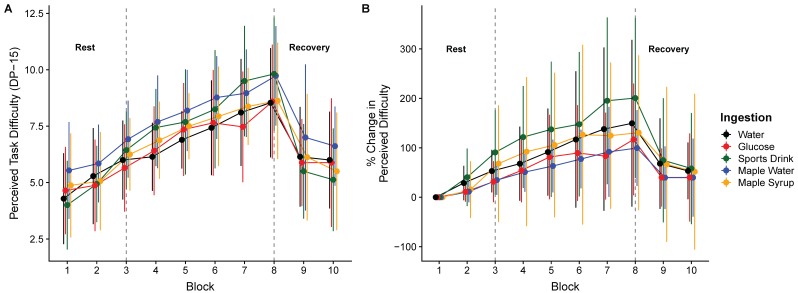
Perceived task difficulty (DP-15) (**A**) and percentage of change (**B**), at the baseline, over the high-intensity intermittent exercise protocol and during recovery (mean ± SD).

The main effect of time was found for both RPE (F_(2147)_ = 180.3, *p* < 0.0001) and DP-15 (F_(3219)_ = 44.3, *p* < 0.0001). No significant interaction was observed for RPE (F_(8147)_ = 1.24, *p* = 0.28) or DP-15 (F_(12,219)_ = 0.60, *p* = 0.84). The results are presented in Figure 3 and Figure 4. Both RPE and DP-15 increased from the second and third HIIE bout, respectively, until the end of the exercise period in all conditions.

**Figure 5:**
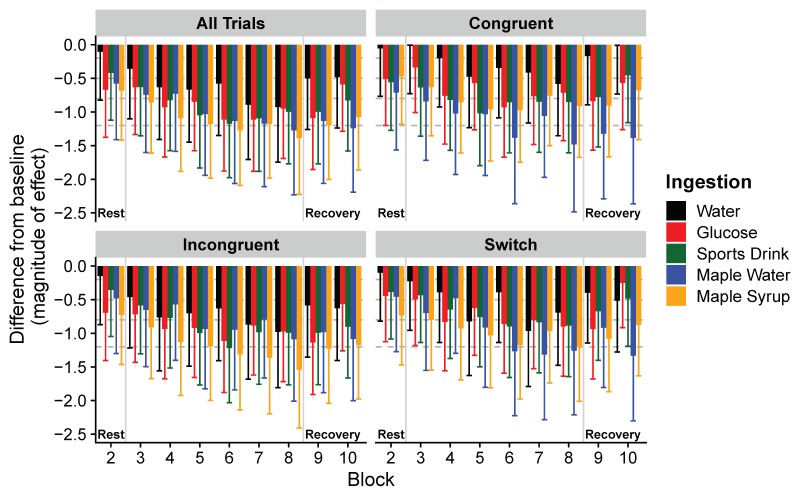
Magnitude of differences (Hedge’s g effect size (ES), with 95% confidence intervals) in reaction times for all trials, for congruent, incongruent and switching trials and for each block compared to baseline. A decrease in ES represents a faster reaction time compared to baseline cognitive performance. Horizontal dashed lines correspond to very small (<0.2), small (0.2 < ES < 0.5), moderate (0.5 < ES < 0.8), large (0.8 < ES < 1.2), and very large (ES > 1.2) effects [41].

Reaction times (RT) and accuracies are presented in Table 2. When water ingestion is compared to glucose, CSD, maple syrup, and maple water ingestion, the ANOVA revealed the main effect of time for RT_total_ (F_(6517)_ = 43.6, *p* < 0.0001), RT_incongruent_ (F_(6440)_ = 39.8, *p* < 0.0001), and for RT_congruent_ (F_(6492)_ = 20.3, *p* < 0.0001). The ANOVA also revealed a main effect on time (F_(6448)_ = 27.3, *p* < 0.0001), and a trend towards ingestion × time interaction (F_(22,448)_ = 1.48, *p* = 0.07) for RT_switch_. In this condition, we found that in the control condition (water), the reaction time was faster than the baseline only in the last bouts of exercise and returned to the baseline in the recovery period. Whenever CHO were ingested (all ingestions except water), we found a decrease in reaction time (faster) from the baseline of the protocol, which remained fast during the post-exercise recovery. Based on the effect size, the magnitude of the decrease in the reaction times during exercise can be considered small to large in the control condition. A small to large decrease in reaction times was found with all CHO solutions ingested during exercise. During the recovery period, reaction times remain shorter than baseline values, and this decrease can be considered to be moderate to large. Only the ingestion of maple products led to a very large decrease in reaction time during the recovery period. Cognitive flexibility performance during the protocol is shown using the magnitudes of effects, which are presented in Figure 5. Concerning the accuracy, there was only a main effect on time (F_(3259)_ = 2.95, *p* = 0.03), and multiple comparisons show that only with the water ingestion did subjects produce more error at the end of the last bout of exercise (*p* < 0.05, ES = 1.0). The large decrease of the reaction time observed at the end of the last bout of exercise with the water ingestion need so to be nuanced since subjects produced more errors. 

**Figure 6:**
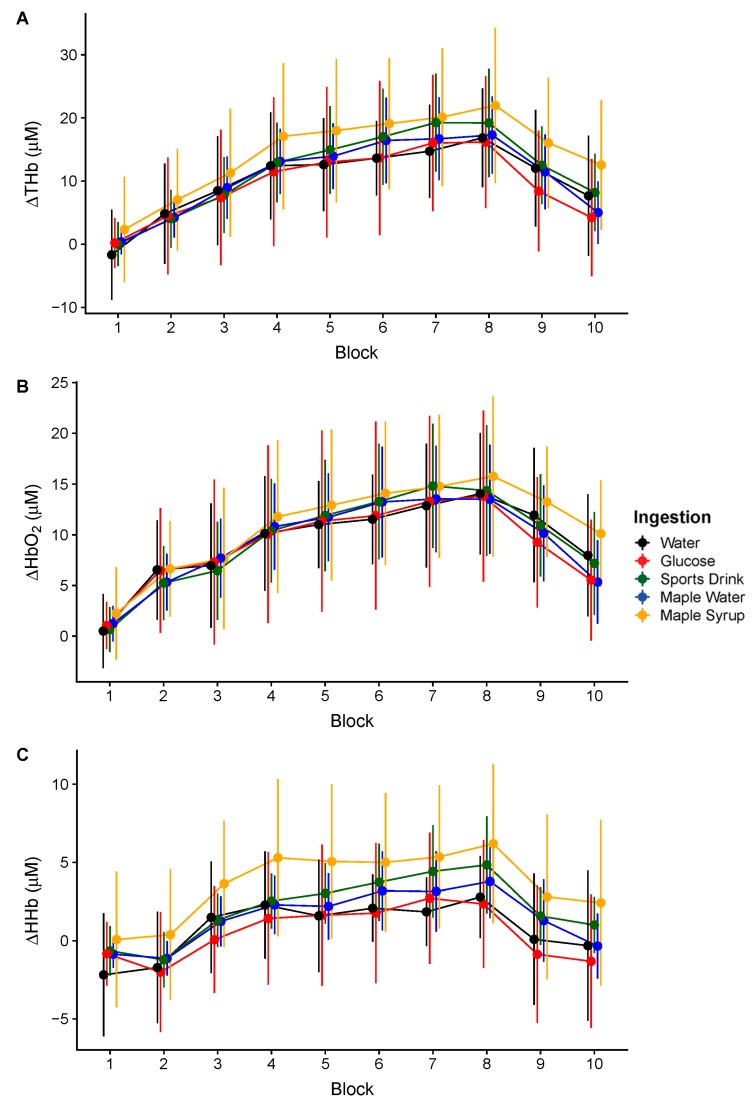
Cerebral oxygenation changes for ThB (**A**), HbO_2_ (**B**) and HHb (**C**), at the baseline, over the high-intensity intermittent exercise protocol and during recovery (means of eight channel ± SD).

Results from cerebral oxygenation showed the main effect of time for ThB (F_(3228)_ = 129.6, *p* < 0.0001), for HHb (F_(4294)_ = 46.0, *p* < 0.0001), and for HbO_2_ (F_(3197)_ = 110.3, *p* < 0.0001). ThB, HbO_2_ and HHb significantly increased starting at the second block and returned towards the baseline after exercise in all conditions. These results are shown in Figure 6.

## 4. Discussion

The aims of this study were to compare the effect of various CHO solutions ingested before and during HIIE on cognitive flexibility, cerebral oxygenation and, more specifically, sports drinks made from diluted maple syrup and concentrated maple water. Our results show that solutions containing CHO, independently of their type, lead to an improvement in cognitive performance, and more specifically on switching trials, during cognitive flexibility tasks, without any observable change in cerebral oxygenation compared to control condition. CHO ingestion before exercise allows for shorter reaction times early-on after the first HIIE bout when compared to baseline, which remain low during post-exercise recovery, whereas the control condition, when only water is ingested, is associated with shorter reaction times during exercise but not during post-exercise recovery. The magnitude of the differences observed when CHO are ingested is greater for the switching trials than in the control condition, which is known to be particularly difficult. This confirms the hypothesis that CHO ingestion could be more beneficial when performing demanding cognitive tasks. In addition, this study shows that maple syrup and maple water, which are unprocessed CHO, appear to increase cognitive performance in a similar way to other common CHO sources (glucose and CSD) during HIIE.

In the current study, CHO ingestion had no significant impact on ratings of perceived exertion (RPE) and of the perceived difficulty of the cognitive task. These results corroborate several studies which reported that CHO does not impact RPE [8]. Results from the literature are however inconsistent: some studies also show that CHO can lead to a decrease in RPE at the same power output [21]. The experimental design in the current study, where all ingestion conditions were independent, could explain why no effect of CHO was observed on perceptual responses. The positive impact of CHO ingestion on RPE is often reported in crossover studies, where the same participants are tested in different experimental conditions. For example, Pomportes et al. [21] reported that the ingestion of glucose or guarana solutions did reduce RPE during exercise. Williams and Rollo [42] also reported that CHO feedings were associated to a lowered perception of effort during intermittent running exercise.

With regard to the effects of exercise on cognitive flexibility, our results show that acute high-intensity exercise temporarily improves cognitive function, but this gain disappears rapidly after the cessation of exercise, as observed in the control condition. These results corroborate previous findings [28,43]. However, this positive effect of HIIE on reaction time is also associated with a lower accuracy towards the end of exercise, probably due at least in part to the central and/or peripheral fatigue induced by HIIE [44,45]. Indeed, under exercise-induced fatigue, cognitive performance can be associated with faster reaction times but also with greater errors [46]. As for the impact of CHO ingestion on cognitive flexibility, the magnitude of the effect was comparable between CHO solutions, but only maple products showed a large to very large effect on reaction time in the switching task, both at the end of the HIIE and during the post-exercise recovery period. Glucose and CSD all show a moderate effect during the recovery period. Also, the impact of CHO ingestion seems to affect reaction times and maintained accuracy throughout the protocol. Usually, the ingestion of CHO is associated with improved reaction times rather than accuracy [21].In our study, the HIIE protocol probably led to greater peripheral and/or central fatigue than the submaximal exercise used in the study by Pomportes et al. [21], and this could explain why the participants in the control condition produced more errors. These results suggest that CHO ingestion allows better-sustained cognitive performance during HIIE.

Only a few studies have reported the effects of acute CHO ingestion on cognitive performance. For example, Collardeau et al. [19] reported choice reaction times before and after running 100 min at 64% of VO_2max_, with or without CHO ingestion (2 mL∙kg^−1^ every 15 min). The results show a significant decrease in reaction time when CHO is ingested (689 ± 51 to 654 ± 63 ms), whereas no significant change is reported in the placebo group (688 ± 104 vs. 676 ± 73 ms). Results from Bottoms et al. [20] are in agreement with these findings and show that the ingestion of a CHO solution (6.4% CHO per volume) significantly improved visual reaction time during simulated squash play. Moreover, Lieberman et al. [22] found that vigilance and mood state were improved when CHO was ingested (6 and 12% CHO per volume) during sustained simulated combat operation in soldiers. However, a few studies have also reported no effect of CHO ingestion on cognitive performance [8]. The reasons for these contradictory results are not entirely clear, but some of the cognitive tests may not be sensitive enough to detect possible changes due to CHO ingestion [8].The CHO dose, type and timing used in the studies, as well as the pre-experimental diet (fasting or not, CHO content, etc.), could also explain these mixed results. In our study, we found that maple syrup and maple water have the same impact as glucose or CSD on cognitive flexibility. Also, most of the studies that examined the impact of CHO on cognitive performance rarely report the participant’s fasting glycemic response, which could lead to disparity in the results if not controlled for.

A few mechanisms were purported to explain the changes in cognitive performance with CHO ingestion. Indeed, the increase in arousal is generally associated with an improvement in cognitive performance, resulting in a decrease in the reaction times [31]. CHO ingestion may also mediate cognition by contributing to an increase in the synthesis of certain neurotransmitters, such as acetylcholine and serotonin [4]. A third mechanism for the cognitive improvements associated with CHO ingestion follow from mouth-rinsing studies. These studies highlight the presence of CHO-sensitive receptors in the mouth with afferent inputs to the central nervous system, and growing evidence from neuroimaging studies confirms these neural pathways [5,6,7]. For example, a greater activation of brain regions associated with reward and motor control (insula/frontal operculum, orbitofrontal cortex and striatum) was measured using functional imaging following mouth rinsing [5,7]. Turner et al. [7] have also shown that a CHO mouth rinse can enhance the activation of neural pathways involved in visual perception. More recently, the activation of the CHO receptors in the mouth was also associated with better cerebral activity using electroencephalography for orbitofrontal and dorsolateral prefrontal cortex [6]. Further evidence supporting an improvement in cognitive performance following CHO ingestion arises from studies on cerebral oxygenation. Gagnon et al. [17] reported a positive impact of ingesting 50 g of glucose on cerebral oxygenation in older adults in dual cognitive tasks. Although results from the current study do not corroborate these findings, the large interindividual variations, the crossover design and the lower dose of CHO administered (60 g) could potentially explain why cerebral oxygenation was not affected by CHO ingestion. Due to the limited number of studies on the effect of CHO on cerebral oxygenation and the contradictory effects noted, more studies will be needed to better understand these responses.

Although the results of this study are interesting and encouraging for the use of maple products on cognitive performance during exercise, there are several limitations to consider. In particular, the lack of effects of CHO on perceptual responses and cerebral oxygenation and the trends observed in the most complex component of our cognitive task may be due to the chosen experimental design (parallel, with independent groups). Although a crossover design is typically preferred to report such comparisons, the multiple experimental conditions in the current study would have required multiple laboratory visits over an extended period. Indeed—and although the results of the literature are contradictory—it seems that the majority of studies with a crossover design report positive effects of glucose ingestion on cognitive performance and perceptual responses during exercise. As the current study shows, interindividual variability in the observed responses, partly due to the same dose of CHO (60 g) being administered to all participants regardless of body weight, could have led to more modest effects. Nevertheless, our results seem to confirm previous hypotheses and are the first to show that maple products can provide similar benefits to more common sources of CHO. Interestingly, our results confirm that for the same cognitive impact, maple products and CSD induce a more modest increase in glycemia than glucose ingestion. This result is the first in humans to confirm the results obtained in animals [24]. The current results are encouraging and open up new perspectives regarding the use of maple products during high-intensity intermittent activities.

## 5. Conclusions

This is the first study which assesses the impact of ingesting different types of carbohydrate (CHO) solution on cognitive flexibility and cerebral oxygenation during high-intensity intermittent exercise (HIIE). This study shows that CHO ingestion, regardless of its type, tends to improve cognitive performance throughout exercise. This study opens up new perspectives concerning the use of maple products to increase our cognitive performance during exercise, since they have the same effect as glucose and sports drinks. Further interventional studies are needed to confirm our findings and determine the impact of maple products on cognitive performance and brain activity. The question of the dose–response relationship also arises, and future research will be needed to confirm the impact of the dose of CHO or maple products on cognitive performance during exercise.

## Figures and Tables

**Table 1:** Subject characteristics.

	Water	Glucose	Sports Drink	Maple Water	Maple Syrup
*n*	16	19	18	14	18
Age (year)	24.4 ± 4.6	30.2 ± 7.6	31.1 ± 6.1	32.7 ± 7.8	25.7 ± 5.0
Mass (kg)	71.6 ± 7.9	76.1 ± 11.6	74.7 ± 8.4	74.3 ± 9.7	69.6 ± 8.9
Height (m)	1.79 ± 0.05	1.81 ± 0.07	1.80 ± 0.10	1.78 ± 0.08	1.77 ± 0.07
VO_2max_ (mL·kg^−1^·min^−1^)	59.9 ± 7.8	56.8 ± 7.2	57.6 ± 7.2	58.7 ± 9.1	63.4 ± 7.1
Maximal power output (W)	301 ± 51	302 ± 51	314 ± 39	313 ± 38	309 ± 47
Plasma glucose concentration (mmol·L^−1^)					
Pre-ingestion	4.99 ± 0.49	5.19 ± 0.56	5.33 ± 0.60	5.21 ± 0.38	5.07 ± 0.48
120 min post-ingestion	5.76 ± 0.97	5.53 ± 1.01	5.55 ± 0.95	5.59 ± 0.67	5.32 ± 1.06

**Table 2:** Reaction times and accuracies on the cognitive task at the baseline (block 1), at rest (block 2), over the high-intensity exercise protocol (blocks 3–8), and during recovery (blocks 9–10).

	Ingestion	*p* Value	Block 1	Block 2	Block 3	Block 4	Block 5	Block 6	Block 7	Block 8	Block 9	Block 10
All trials (ms)	Water	C: 0.22	1017 ± 298	990 ± 282	922 ± 237	841 ± 252 *	832 ± 252 *	847 ± 286 *	785 ± 209 *	787 ± 178 *	885 ± 226	890 ± 227
	Glucose		1116 ± 284	952 ± 195 *	967 ± 172 *	899 ± 161 *	897 ± 224 *	856 ± 161 *	834 ± 210 *	858 ± 253 *	853 ± 181 *	928 ± 348 *
	Sports drink	T < 0.0001	1156 ± 380	1026 ± 225	956 ± 240 *	909 ± 180 *	847 ± 160 *	815 ± 141 *	817 ± 209 *	856 ± 184 *	847 ± 211 *	897 ± 220 *
	Maple water		1284 ± 405	1082 ± 276 *	1020 ± 284 *	1030 ± 269 *	941 ± 221 *	916 ± 195 *	883 ± 245 *	868 ± 201 *	918 ± 191 *	873 ± 216 *
	Maple syrup	I: 0.25	1092 ± 332	896 ± 226 *	857 ± 194 *	791 ± 195 *	764 ± 201 *	746 ± 182 *	767 ± 195 *	721 ± 171 *	764 ± 190 *	791 ± 206 *
Incongruent trials (ms)	Water	C: 0.13	1026 ± 318	983 ± 289	896 ± 237	808 ± 242 *	816 ± 272 *	829 ± 301 *	781 ± 230 *	765 ± 193 *	861 ± 238 *	853 ± 221 *
	Glucose		1126 ± 286	955 ± 199 *	954 ± 178 *	902 ± 176 *	888 ± 226 *	860 ± 173 *	899 ± 218 *	854 ± 268 *	846 ± 192 *	928 ± 405 *
	Sports drink	T < 0.0001	1157 ± 405	1039 ± 254	957 ± 260 *	906 ± 210 *	845 ± 169 *	787 ± 124 *	835 ± 213 *	840 ± 190 *	835 ± 206 *	869 ± 191 *
	Maple water		1260 ± 442	1080 ± 281 *	1010 ± 297 *	1039 ± 313 *	924 ± 230 *	924 ± 214 *	937 ± 349 *	868 ± 231 *	911 ± 218 *	864 ± 246 *
	Maple syrup	I: 0.48	1073 ± 305	876 ± 228 *	841 ± 185 *	779 ± 198 *	757 ± 210 *	734 ± 190 *	725 ± 184 *	695 ± 154 *	747 ± 209 *	760 ± 214 *
Congruent trials (ms)	Water	C: 0.50	993 ± 283	1007 ± 325	992 ± 301	933 ± 341	872 ± 221	895 ± 290	879 ± 268	846 ± 213	950 ± 243	990 ± 347
	Glucose		1087 ± 313	944 ± 244	1002 ± 184	891 ± 183 *	924 ± 254 *	846 ± 183 *	878 ± 221 *	870 ± 292 *	873 ± 172 *	928 ± 244
	Sports Drink	T < 0.0001	1154 ± 366	990 ± 195	954 ± 253 *	913 ± 187 *	852 ± 194 *	887 ± 240 *	895 ± 224 *	897 ± 210 *	884 ± 320 *	981 ± 398
	Maple water		1334 ± 381	1087 ± 301 *	1039 ± 305 *	1012 ± 217 *	976 ± 293 *	894 ± 221 *	1001 ± 215 *	875 ± 198 *	933 ± 177 *	900 ± 209 *
	Maple syrup	I: 0.20	1143 ± 479	953 ± 306 *	902 ± 248 *	824 ± 212 *	784 ± 212 *	778 ± 212 *	850 ± 253 *	790 ± 252 *	811 ± 189 *	885 ± 237 *
Switching trials (ms)	Water	C: 0.40	1035 ± 302	1008 ± 276	972 ± 264	916 ± 306	819 ± 206 *	914 ± 321	794 ± 172 *	842 ± 247 *	928 ± 232	890 ± 252
	Glucose		1124 ± 303	998 ± 263	1000 ± 184	918 ± 171 *	944 ± 269 *	912 ± 164 *	899 ± 252 *	873 ± 246 *	876 ± 213 *	1035 ± 414 *
	Sports drink	T < 0.0001	1160 ± 451	1028 ± 187	1002 ± 251	933 ± 199 *	890 ± 217 *	850 ± 177 *	865 ± 204 *	853 ± 179 *	910 ± 265 *	974 ± 306 *
	Maple water		1282 ± 381	1131 ± 266	1032 ± 320 *	1111 ± 327	971 ± 280 *	893 ± 190 *	874 ± 198 *	888 ± 207 *	995 ± 207 *	887 ± 152 *
	Maple syrup	I: 0.07	1190 ± 459	916 ± 252 *	898 ± 225 *	849 ± 231 *	811 ± 229 *	770 ± 196 *	827 ± 253 *	763 ± 184 *	802 ± 200 *	854 ± 278 *
Accuracy (%)	Water	C: 0.43	0.97 ± 0.03	0.95 ± 0.04	0.95 ± 0.04	0.94 ± 0.05	0.95 ± 0.05	0.93 ± 0.09	0.94 ± 0.08	0.91 ± 0.07 *	0.95 ± 0.09	0.92 ± 0.11
	Glucose		0.96 ± 0.05	0.96 ± 0.05	0.97 ± 0.04	0.96 ± 0.05	0.95 ± 0.05	0.95 ± 0.06	0.96 ± 0.06	0.94 ± 0.08	0.95 ± 0.08	0.94 ± 0.09
	Sports drink	T: 0.03	0.93 ± 0.09	0.94 ± 0.04	0.95 ± 0.07	0.92 ± 0.08	0.93 ± 0.06	0.98 ± 0.08	0.90 ± 0.11	0.90 ± 0.11	0.92 ± 0.12	0.91 ± 0.12
	Maple water		0.96 ± 0.03	0.96 ± 0.02	0.97 ± 0.04	0.95 ± 0.04	0.95 ± 0.06	0.95 ± 0.06	0.95 ± 0.08	0.94 ± 0.09	0.94 ± 0.11	0.95 ± 0.11
	Maple syrup	I: 0.90	0.96 ± 0.04	0.95 ± 0.05	0.96 ± 0.04	0.95 ± 0.05	0.94 ± 0.07	0.94 ± 0.07	0.94 ± 0.07	0.95 ± 0.06	0.96 ± 0.06	0.96 ± 0.07

* Different from baseline (Block 1); C: ingestion (main effect); T: time (main effect); I: interaction (ingestion × time).
